# Ecoinformatics Reveals Effects of Crop Rotational Histories on Cotton Yield

**DOI:** 10.1371/journal.pone.0085710

**Published:** 2014-01-17

**Authors:** Matthew H. Meisner, Jay A. Rosenheim

**Affiliations:** 1 Department of Evolution and Ecology, University of California Davis, Davis, California, United States of America; 2 Department of Statistics, University of California Davis, Davis, California, United States of America; 3 Department of Entomology and Nematology, University of California Davis, Davis, California, United States of America; Federal University of Viçosa, Brazil

## Abstract

Crop rotation has been practiced for centuries in an effort to improve agricultural yield. However, the directions, magnitudes, and mechanisms of the yield effects of various crop rotations remain poorly understood in many systems. In order to better understand how crop rotation influences cotton yield, we used hierarchical Bayesian models to analyze a large ecoinformatics database consisting of records of commercial cotton crops grown in California's San Joaquin Valley. We identified several crops that, when grown in a field the year before a cotton crop, were associated with increased or decreased cotton yield. Furthermore, there was a negative association between the effect of the prior year's crop on June densities of the pest *Lygus hesperus* and the effect of the prior year's crop on cotton yield. This suggested that some crops may enhance *L. hesperus* densities in the surrounding agricultural landscape, because residual *L. hesperus* populations from the previous year cannot continuously inhabit a focal field and attack a subsequent cotton crop. In addition, we found that cotton yield declined approximately 2.4% for each additional year in which cotton was grown consecutively in a field prior to the focal cotton crop. Because *L. hesperus* is quite mobile, the effects of crop rotation on *L. hesperus* would likely not be revealed by small plot experimentation. These results provide an example of how ecoinformatics datasets, which capture the true spatial scale of commercial agriculture, can be used to enhance agricultural productivity.

## Introduction

Maximizing agricultural crop yield is an important goal for several reasons. First, a growing worldwide population will generate increased demand for agricultural resources [1]. Since expanding the land area devoted to agriculture is often unfeasible, or would involve the destruction of sensitive landscapes such as forests and wetlands, the only way to meet this demand will be to increase the crop yield generated from existing farmland. Second, there are substantial economic incentives for profit-seeking farmers to maximize the yield of their crops, especially given the low profit margins typical of commercial agriculture [2].

Farmers make a wide range of decisions regarding the management of their crops, involving pest management, planting/harvest dates, fertilization, irrigation, and, as we focus on in this study, crop rotation. These decisions are, along with external factors that fall outside farmers' control, such as weather, likely to affect crop performance and yield substantially. A rigorous quantitative understanding of the factors, including farmer management decisions, that affect crop yield is an essential prerequisite for developing management strategies that maximize yield.

A critical factor known to affect crop yield in a given field is the crop rotational history of that field [3]. There are several possible mechanisms by which the crops previously grown in a field can affect crop yield. First, different crops have different effects on the nutrient composition of the soil, so the identities of crops previously grown in a field can affect nutrient availability and crop yield [3]. For example, nitrogen-limited crops can benefit from rotation with nitrogen-fixing legumes [4], and phosphorus nutrition in California cotton is shaped by whether or not the previous crop received phosphorus fertilizer [5]. Second, certain crops may increase the local abundance of particular insect pests and pathogens [6]–[8]. Since different crops are often susceptible and resistant to different pathogens and pests, the identities of the crops recently grown in a field can affect yield. For example, if one crop increases local abundances of an insect pest that also attacks a second crop, planting the second crop immediately following the first may lead to decreased yield resulting from attack from the built up local pest population. In contrast, such a yield depression could potentially be averted if the second crop were planted following a crop that does not lead to local accumulation of the pest. In monocultures of wheat, substantial yield declines have been noted and attributed to the buildup of the soil-borne fungal pathogen *Gaeumannomyces graminis* [9]. Third, many studies have shown that a field's crop rotational history can strongly affect weed densities [10]. Numerous other mechanistic explanations for the yield effects of crop rotation have also been suggested [3].

Crop rotation has been practiced for thousands of years; evidence for its inception dates back to ancient Roman and Greek societies [11], [12]. Experimental studies on the effects of crop rotation first appeared in the early 20th century, revealing that growing crops in rotation led to increased crop yields of up to 100% compared to continuous planting of a single crop [13], [14]. Interest in the yield effects of crop rotation waned during the middle of the 20th century, due to the increasing availability of cheap fertilizers, insecticides, and herbicides [3], [14]. However, crop rotation continues to be a relevant and important practice; low-input farming remains desirable due to the costs of fertilizers and pesticides, and fertilizer and pesticide applications can often not fully compensate for the benefits afforded by crop rotation [3]. In addition, the significant environmental and public health concerns surrounding fertilizer and pesticide use [1], [15] highlight the desirability of methods of increasing crop yield through alternative methods such as crop rotation.

The effects of rotational histories on yield are well understood for some crops, such as corn, where rotation is recognized to be crticial in avoiding the buildup of corn rootworms [16]. However, for many crops, the direction, magnitude, and mechanism of the effect of crop rotational histories on crop yield remain poorly understood [3]. Cotton is one such crop. Experimental field studies of the effect of crop rotation on cotton yield have demonstrated increased cotton yield, compared to continuous cultivation of cotton, when cotton is grown in rotation with sorghum [17], [18], corn [19], and wheat [20], [21]. Despite these useful results, only a small subset of possible rotations has been studied, experiments have been restricted to plots significantly smaller than typical commercial cotton fields, and mechanisms for these effects remain poorly understood. To help address these limitations, we seek to expand upon this work by exploring the effects of crop rotational histories on yield in commercial cotton fields in California, using an “ecoinformatics” approach [22] capitalizing on existing observational data gathered by growers and professional agricultural pest consultants.

In recent years, there has been a surge in research and interest involving the rapidly emerging field of “big data.” The big data movement has been fueled by several developments, including a dramatic increase in the magnitude of data generation, an improved ability to cheaply store, manipulate, and explore massive datasets, and the development of new analytic methods [23]. Most importantly, the movement has been driven by a growing realization that existing data, and data generated as a byproduct of our everyday lives, can be leveraged to explore key questions about nature and human behavior, even if the data were not collected for this purpose [24]. Ecoinformatics is a nascent field focused on harnessing the power of big data to address questions in environmental biology. Ecoinformatics approaches typically involve the analysis of large datasets, the synthesis of diverse data sources, and the analysis of pre-existing, observational datasets [22]. In some commercial agricultural settings, farmers, along with hired consultants, collect a great deal of regular data about their fields that are used to guide real-time crop management decisions, such as the timing of pesticide applications. By capitalizing on data that are already generated as a byproduct of commercial agriculture, ecoinformatics provides a low-cost means of obtaining a large dataset that can be used to explore key questions in agricultural biology, some of which might be too difficult or too costly to explore experimentally. Furthermore, the large size of datasets created for ecoinformatics can afford greater statistical power than could possibly be generated through experimental work.

Experimentally studying the yield effects of crop rotational histories is challenging for several reasons. There are a plethora of possible rotational histories, which means that a large number of treatments would be required to explore the space of possible rotational histories thoroughly. Furthermore, experimentally studying effects of crop rotations requires experiments spanning several growing seasons, which may be logistically challenging. Finally, in order to maintain realism and applicability to commercial fields, which are typically quite large, sizeable experimental plots would be required, especially in light of research suggesting that landscape composition as far as 20 km from a focal field can affect the densities of agricultural pests in that field [25]. While yield effects of non-mobile factors such as soil characteristics may be readily detected through small plot experimentation, the effects of highly mobile arthropods may only be detected at much larger spatial scales.

An ecoinformatics approach offers attractive solutions to these challenges. Since we analyze a large preexisting dataset that includes over a thousand records, a diversity of the possible crop rotational histories already exists in the dataset. In addition, our dataset spans 11 years of data, so the data span the temporal scale necessary to ask questions regarding effects of multi-year rotational histories. And, since the data come from the exact setting where we wish to apply our results, the data are realistic and capture the appropriate spatial scale of commercial agriculture.

First, we sought to identify which crop rotational histories are associated with increased and decreased cotton yield, and to quantify these yield effects. We then explored possible explanations for the yield effects identified in the previous step by examining the associations between crop rotational histories and pest abundance.

## Materials and Methods

### Dataset

The dataset was constructed by collecting existing crop records from commercial cotton fields in California's San Joaquin Valley. The data were shared by both growers and pest control advisors (PCAs), professional consultants hired to monitor field conditions and provide crop management recommendations. The dataset contains records of 1498 unique field-year instances from 566 unique fields, ranging from 1997 to 2008. Growers and PCAs collect and maintain detailed records of the conditions in their fields; numerous variables were recorded for each field-year record, and the following were used in our analyses:

1. Cotton yield. Measured once for each field-year instance, cotton lint yield was measured in bales/acre (converted to kg/ha for our analyses) and recorded for 1240 of the 1498 total records.
2. Crop rotational histories. The identity of the crop grown in the same field in previous growing seasons was recorded. For some fields, records extended back for 10 years. However, the vast majority of fields did not have records extending this far into the past. There were 15 unique crops that appeared in rotational histories: alfalfa, barley, carrots, corn, cotton, garbanzo beans, garlic, lettuce, melons, onions, potatoes, safflower, sugarbeets, tomatoes, and wheat.
3. Surrounding crops. For 1026 of the 1498 crops, we had data on the identity of the crop grown in each of the 8 fields immediately adjoining the focal field (to the North, Northeast, East, Southeast, South, Southwest, West, and Northwest).
4. Cotton variety. The database consisted of records of two different cotton species: *Gossypium barbadense* L. (“Pima cotton”) and *Gossypium hirsutum* L. (“upland cotton”).
5. *Lygus hesperus* densities. The plant bug *L. hesperus* is one of the most damaging pests of cotton, and a frequent target of insecticide applications [26], [27]. PCAs measured *L. hesperus* densities approximately weekly, primarily during June and July. The PCAs' sampling procedure consisted of 50 swings of a sweep net across the top of the plant canopy. Since not all PCAs sampled on the same days or at exactly the same intervals for all fields, we transformed successive samples into mean *L. hesperus* density estimates by calculating the area under the linear curve of *L. hesperus* density versus time and dividing by the number of days in the sampling interval.

### Modeling approach

We employed a hierarchical Bayesian modeling approach, fitting linear mixed models to explore our questions about the effects of crop rotational histories on cotton yield. Mixed models combine the use of random effects and fixed effects, making them ideally suited for analysis of data that are structured, or clustered, in some known way, such that separate observations from within clusters are expected to be similar to one another [28]. When we model a source of clustering using a random effect, we assume that each cluster-specific parameter was drawn from a common distribution, and we estimate the parameters of this distribution from the data. We use this common distribution as the prior when calculating the posterior distribution of each cluster-specific parameter. The parameters (often called hyperparameters) of the distribution of cluster-specific parameters have posteriors that are estimated from the data, typically after assuming uninformative priors for the hyperparameters [28]. Using a common, empirical prior for all cluster-specific parameters allows pooling of information across clusters, so that data from all clusters can help inform estimates of every other per-cluster parameter. Assuming all clusters are the same introduces high bias and tends to underfit the data, whereas estimating fixed effects for each cluster introduces high variance and tends to overfit the data; however, using a random effect provides an optimal compromise between introducing bias and introducing variance [28]. In this dataset, there are several plausible sources of clustering.

1 First, we expect the data to be clustered by field, since there likely exist field-specific factors that affect yield, such as soil characteristics, local climate, and grower agronomic and pest management practices. We controlled for variable yield potential between fields by including field identity as a random effect in our models. Random effects allow pooling of information across clusters, so they are particularly useful when there are few observations from some clusters - a situation in which it is difficult to accurately estimate each per-cluster parameter with only the data from that one cluster [28]. Since there are three or fewer records for 78% of the fields in our database, we feel that including field as a random effect was preferable to trying to estimate field-specific fixed effects with very few observations per field.

     Additionally, including field as a random effect provides a straightforward way to make predictions for fields not represented in our database. Since modeling field as a random effect involves estimating a distribution of per-field parameters, we can simply sample a field-specific parameter from this distribution if we wish to make predictions about a previously unobserved field. Uncertainty in this field-specific parameter can be propagated by simulating many samples from this distribution, while simultaneously accounting for uncertainty in the parameters of this distribution. However, if we were to model field as a fixed effect, we would not estimate a distribution of field-specific parameters. We would only estimate parameters for the specific fields in our database, leaving us with no obvious way to make inferences about new fields.

2 Second, we expect that our data are clustered by year, since there is substantial between-year variability in climate, particularly in the winter and early spring. Climatic variables can affect crop performance, planting date, and insect pest populations, all of which can in turn affect cotton yield. To control for and quantify variation in yield due to year-specific factors, we included year as a random effect in our models. Our reasons for including year as a random effect are the same as those for field: there are few observations from some years, and we may wish to make predictions for future years not covered by the existing database.

All models were fit using a No-U-Turn Sampler variant of Hamiltonian Markov Chain Monte Carlo [29] implemented in Stan version 1.3.0, accessed through the rstan packing in R [30], [31]. We ran three chains from random initializations, each with 10,000 samples, and discarded the first 5,000 samples from each as burn-in. Inferences were based upon the remaining 15,000 samples. We checked convergence by making sure that 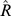, an estimate of the potential scale reduction of the posterior if sampling were to be infinitely continued, was near 1 [32].

### Models

#### Model 1

To explore the yield effects of the crop grown in the same field the previous year, we fit a linear mixed model with yield as the response variable. The predictor variable of primary interest was the identity of the crop grown in that field the previous year, which was included as a fixed effect.

Given that we are working with an observational dataset, a critical step in order to make meaningful inferences about the variable of primary interest - the crop grown the year before - was to control, to the extent possible, for potentially confounding variables that could generate spurious correlations and taint the validity of our inferences about crop rotation. To control for variable yield potential between fields and years, field and year were included in the model as random effects. The field terms control for the possibility that some fields may have higher yield potential due to their location, soil characteristics, or growing practices; the year terms control for the substantial year-to-year variation in cotton yield, which likely results from yearly weather differences. A term indicating cotton species (Pima or upland) was included in the model to account for yield differences between cotton species. Cotton species was modeled as a fixed effect, since there are only two possible categories - not enough to meaningfully estimate a random effects distribution [28]. We also included 15 real-valued fixed effect predictor variables that indicate the number of fields, out of the 8 surrounding fields, planted with each of the 15 crops we analyzed. The goal was to control for effects of the surrounding landscape, and thereby avoid spurious correlations between rotational history (which may be correlated with the crops surrounding the focal crop) and yield.

Our Bayesian modeling approach required the specification of priors for all parameters whose posteriors were estimated using MCMC. Noninformative priors (normal distributions with mean 0 and standard deviation of 100) were used for all fixed effects. The random effects for both field and year were assumed to follow a normal distribution with mean 0 (allowing means of these distributions to be estimated from the data would lead to nonidentifiability with the fixed effects for prior crop identity) and variance hyperparameters estimated from the data. Since the support of variance parameters is constrained to positive real numbers, noninformative inverse gamma distributions with shape and scale parameters set to 0.001 were used as the prior for the variance parameter of the top-level stochastic node, and as the priors for the variance hyperparameters of the field and year random effects distributions.

#### Model 2

To help us understand whether any effects of the crop grown in the field the previous year on cotton yield could be due to effects on *L. hesperus*, we fit the same model as Model 1, but with average June *L. hesperus* abundance as the response variable.

#### Model 3

Next, to formally assess whether there was an association between the effects of crop rotation on yield and the effects of crop rotation on *L. hesperus* abundance, we performed a linear regression of the estimated effects on yield (measured as the posterior means from Model 1) against the estimated effects on *L. hesperus* abundance (measured as posterior means from Model 2). Noninformative 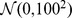 priors were used for the mean and intercept, and a noninformative inverse gamma distribution with shape and scale parameters set to 0.001 was used as the prior for the variance.

#### Model 4

A great deal of experimental evidence has demonstrated that crop rotation leads to increased yield compared to successive plantings of a single crop [3]; therefore, we explored whether or not a yield loss was incurred by cotton crops grown in fields where cotton was grown in previous years. For the 782 fields that had complete crop rotational records for the previous 4 years, we calculated the number of consecutive cotton plantings (from 1 to 4) in the 4 years preceding the focal cotton crop. We then fit a model, with yield as the response variable, using the number of consecutive prior cotton plantings as a predictor (again with the same noninformative prior of 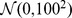). Field, year, and cotton type were included as they were in Models 1 and 2. Since the number of prior consecutive cotton plantings could be correlated with the number of cotton fields in the surrounding landscape during the focal year, we avoided a possible spurious correlation between consecutive cotton plantings and yield by also including a fixed effect for the number of cotton fields in the 8 fields adjacent to the focal field. We chose not to explore rotational histories of specific crops (and instead just grouped all crops into “cotton” or “not cotton”) for longer than one previous year, since the number of possible rotational histories becomes very large and the number of records for each possible history becomes too small to allow for robust statistical analysis.

#### Model 5

To see if the number of consecutive years of cotton cultivation preceding the focal year was associated with June *L. hesperus* densities, we fit the same model as Model 4, but with June *L. hesperus* as the response variable.

## Results

### Model 1

**Figure 1:**
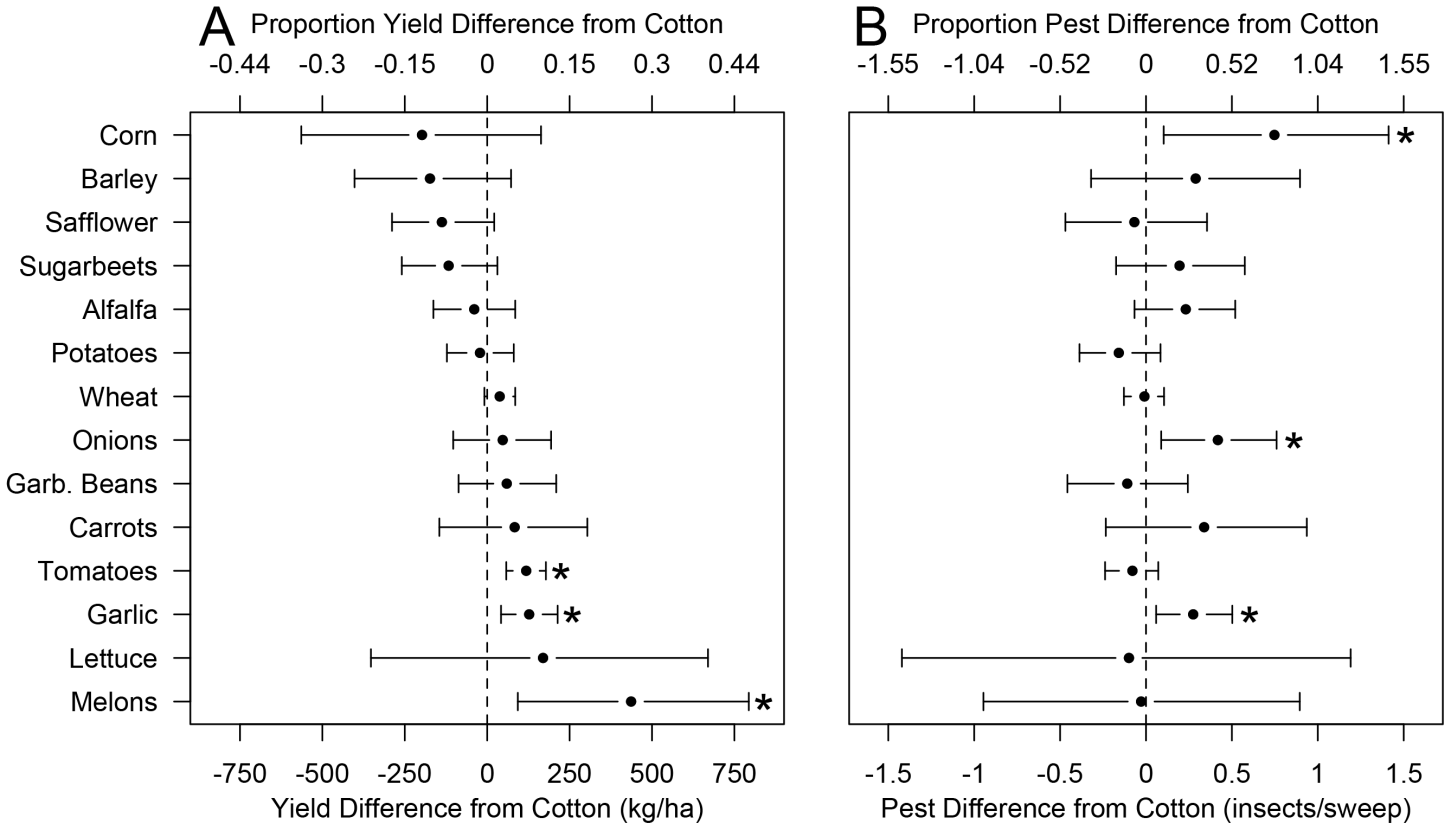
Means and 95% HPDIs of the differences in mean yield (A) and mean June *L. hesperus* density (B) between fields where a certain crop was grown the previous year and where cotton was grown the previous year. 95% HPDIs that do not overlap 0 are marked with a (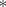).

Using our samples from the joint posterior of Model 1, we calculated, for each crop other than cotton, the posterior distribution of the difference in mean cotton yield in fields where that crop was grown the year before compared to mean yield in fields where cotton was grown the year before. The posterior means of these comparisons, as well as 95% highest posterior density intervals (HPDIs), are displayed in Figure 1A. Highest posterior density intervals are a Bayesian analogue of frequentist confidence intervals; they denote the narrowest region of parameter space containing 95% of the posterior probability [28]. Three crops had 95% HPDIs that did not overlap 0. Garlic (lower limit  = 42.0 kg/ha, upper limit = 213.7 kg/ha), tomatoes (57.9 kg/ha 178.1 kg/ha), and melons (92.9 kg/ha, 793.7 kg/ha) had entirely positive 95% HPDIs, suggesting that previous cultivation of these crops was associated with increased cotton yield. While no crops had entirely negative 95% HPDIs, the posterior probability of safflower and sugarbeets having negative effects on yield was 96% and 95%, respectively, suggesting that cultivation of these crops the previous year was associated with decreased cotton yield. Yield was 153.0 kg/ha higher, with a 95% HPDI of (115.0 kg/ha, 192.8 kg/ha), for upland cotton than for Pima cotton.

### Model 2

Using the joint posterior of Model 2, we calculated the posterior distribution of the difference in mean June *L. hesperus* densities between fields where cotton was the year grown before and where other specific crops were grown the year before. The posterior means of these comparisons, as well as 95% HPDIs, are displayed in Figure 1B. Corn (0.10 insects/sweep, 1.41 insects/sweep), onions (0.09 insects/sweep, 0.76 insects/sweep), and garlic (0.06 insects/sweep, 0.50 insects/sweep) all had 95% HPDIs that were entirely positive, suggesting that previous cultivation of these crops was associated with increased *L. hesperus* abundance. June *L. hesperus* density was 0.35 insects/sweep lower, with a 95% HPDI for this decrease of (0.26 insects/sweep, 0.44 insects/sweep), for upland cotton than for Pima cotton.

### Model 3

**Figure 2:**
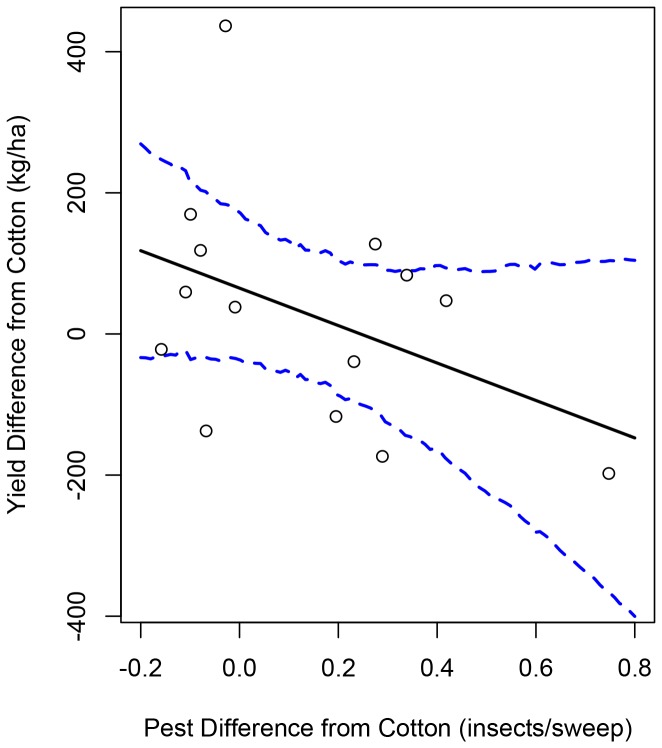
For each of the 14 crops other than cotton, we calculated the posterior mean of the mean difference in yield when that crop was grown in the field the year before compared to when cotton was grown in the field the year before (y-axis; these estimates are also displayed in Figure 1A). We also calculated the posterior mean of the difference in mean June *L. hesperus* densities between fields where a specific crop was grown the year before and where cotton was grown the year before (x-axis; these estimates are also displayed in Figure 1B). These estimates are plotted above (open circles). Then, we fit a linear model by regressing the mean yield differences on the mean *L. hesperus* differences. The posterior mean of the model fit (solid black) and 95% HPDI (dashed blue) are overlaid.

While there were exceptions, we noticed that there was a trend for crops associated with increased pest abundances to also be associated with decreased yield. To more rigorously quantify this trend, for the 14 crops other than cotton, we regressed the posterior mean of the yield difference from cotton against the posterior mean of the *L. hesperus* difference from cotton. There was a negative slope with posterior mean −0.49 and 95% HPDI of (−1.16, 0.15) that marginally overlapped 0; the posterior probability of there being a negative slope was 93.4%. This provided evidence that crops associated with increased June *L. hesperus* densities were also associated with negative effects on yield (Figure 2).

### Model 4

Model 4 suggested that every additional consecutive year of prior cotton cultivation in a field led to reduced cotton yield. Figure 3A displays the posterior distribution of the change in yield for each additional year that cotton was consecutively grown in the field prior to the focal year; the posterior mean for this change in yield was −40.9 kg/ha, with 95% HPDI (−57.5,−23.4 kg/ha). This translates to a mean of the percentage change in yield of −2.4% per year and 95% HPDI of (−1.4%,−3.4%) per year. We refit Model 4 without the term for consecutive cotton plantings; boxplots of the residuals are plotted against consecutive cotton plantings in Figure 3B, where a decreasing trend can be observed. Yield was 169.2 kg/ha higher, with a 95% HPDI of (123.4 kg/ha, 211.5 kg/ha), for upland cotton than for Pima cotton.

**Figure:**
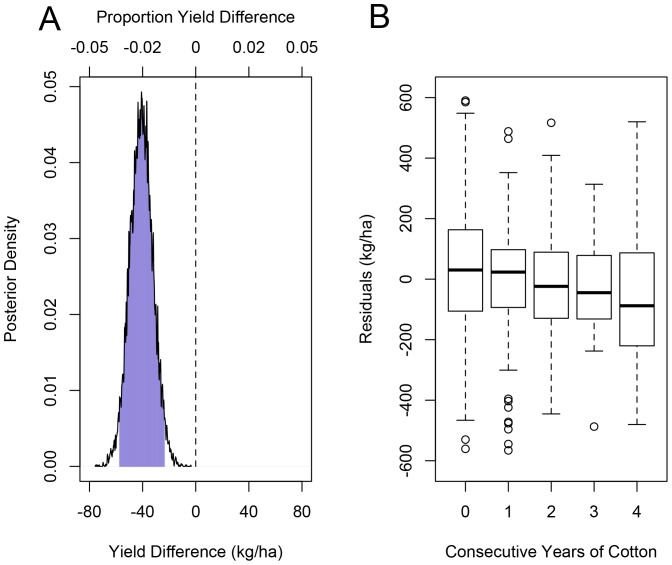
The posterior distribution of the change in yield for every additional year that cotton was grown consecutively in a field prior to the focal year (A). We refit the model without consecutive cotton plantings in the model and display boxplots of the residuals vs. consecutive cotton plantings, where a decreasing trend can be observed (B).

### Model 5

Model 5 revealed a positive association between the number of preceding consecutive cotton plantings and June *L. hesperus* densities; the posterior mean of the slope regressing June *L. hesperus* on consecutive cotton plantings was 0.037 insects/sweep with a 95% HPDI that slightly overlapped 0 of (−0.007,0.079). The posterior probability of there being a positive relationship between consecutive cotton plantings and *L. hesperus* densities was 95.3%. June *L. hesperus* density was 0.32 insects/sweep lower, with a 95% HPDI of (0.20 insects/sweep, 0.43 insects/sweep), for upland cotton than for Pima cotton.

## Discussion

Capitalizing on a large existing set of crop records from commercial cotton fields in California, we employed an ecoinformatics approach to explore the effects of crop rotational histories on cotton yield. Our hierarchical Bayesian analyses revealed evidence that several crops, when grown in the same field the year before the focal cotton planting, were associated with either decreased or increased cotton yield (Figure 1A), and either increased or decreased early season densities of the pest *L. hesperus* (Figure 1B). Furthermore, crops associated with decreased yield were generally also associated with increased *L. hesperus* densities, while those associated with increased yield were also associated with decreased *L. hesperus* densities (Figure 2).

These results suggest a possible mechanism for the observed yield effects of these rotational histories. Since *L. hesperus* preferentially attacks certain crops [33], a field cultivated with a crop that is heavily attacked by *L. hesperus* may, if *L. hesperus* disperse from the focal field, increase the abundance of *L. hesperus* in nearby fields. These populations may subsequently attack the crop planted in the focal field the following year, explaining the increase in early-season *L. hesperus* densities that we detected following certain crops. In turn, these increased *L. hesperus* populations may exert strong herbivorous pressure on focal cotton crops, possibly explaining the corresponding decrease in yield.

We believe that the effect of rotational history on early-season *L. hesperus* likely operates at a landscape scale that is larger than the within-field scale. If cotton was grown in a field the previous year, then farmers in the San Joaquin Valley are required to maintain a 90-day plant-free period prior to 10 March of the following year [27]. This prevents *L. hesperus*, which overwinter as adults on live host plants, from overwintering in a focal field where cotton was grown the year before. If a crop other than cotton was grown the previous year, then it could be possible for *L. hesperus* to overwinter in the focal field on residual plant or weed populations; however, since fields are completely plowed prior to planting cotton in the spring, *L. hesperus* adults would still need to temporarily leave the focal field. Therefore, we believe that the preferred host crops for *L. hesperus* increase *L. hesperus* populations at a landscape scale. Then, when cotton, another target of *L. hesperus*, is planted in the same field the following year, the cotton field is attacked by this regional population. If regional populations are already large due to lingering effects from crops grown the previous year, *L. hesperus* populations may move into cotton early in the growing season; this could be particularly damaging given research suggesting that cotton yield is particularly sensitive to *L. hesperus* densities early in the growing season [34]. Using our data, we were not able to determine at exactly what scale the effects of rotation on *L. hesperus* likely operate. We do not believe a within-field scale is plausible, but determining a more precise spatial scale for these effects could be an interesting topic for future research.

Our findings match expectations of crop yield effects based on previous research on *L. hesperus* host crop preferences, lending support to our hypothesis that yield effects of crop rotational histories are, at least partially, mediated by effects on *L. hesperus*. Alfalfa and sugarbeets, both crops for which we found negative effects on yield and positive effects on *L. hesperus* when grown in a field the previous growing season, are all considered preferred hosts for *L. hesperus* [27], and have been shown to also increase *L. hesperus* populations in nearby cotton fields during an individual growing season [33], [35]. Presumably, this effect is due to these crops supporting large *L. hesperus* populations. Large *L. hesperus* populations are known to build up in alfalfa [36], and their dispersal following alfalfa harvesting can threaten nearby cotton crops [27], [37]. *L. hesperus* is also known to emigrate to nearby cotton fields when safflower begins to dry in mid-summer [38]. While the potential for nearby alfalfa [27], [33] and safflower [33], [35] fields to increase *L. hesperus* populations in cotton fields in a given year has been recognized, our results are the first indication that these landscape effects may extend temporally, affecting *L. hesperus* populations, and yield, in the next growing season. Tomatoes, associated with increased yield and decreased pest abundance in our data, have likewise been shown to decrease *L. hesperus* abundances in nearby cotton fields within a given year [33].

While previous experimental work has examined the effects of crop rotations on cotton yield [14], [17], [18], [20], [21], our work expands on these studies in several ways. First, we explore a much wider diversity of possible crop rotational histories, providing quantitative estimates of the cotton yield effects of cultivating 14 different crops the previous year. Second, since we analyze records from commercial cotton fields, our data have the potential to capture yield effects (such as those due to highly mobile arthropods) that could only be detected at this realistic spatial scale. Third, since we have collected data on pest abundances, not only yield, we have also been able to use our data to generate and build evidence for a hypothesized mechanistic explanation of the yield effects we identify.

We also found that farmers incurred a decline in cotton yield of about 2.4% for every additional year cotton was grown consecutively in a field preceding the focal season (Figure 3). This is consistent with previous research suggesting that continuous cultivation of cotton in the same location can reduce yield compared to interspersing cotton with other crops [14], [17], [18], [20], [21]. We also found some evidence that the number of years cotton was grown consecutively in a field was associated with higher June *L. hesperus* densities: the posterior probability of there being a positive association was about 95%. Identifying the actual mechanism underlying this yield effect is beyond the scope of this study, but would be an interesting avenue for future research. It is possible that the yield decline is not caused by changes in *L. hesperus* densities, and instead results from the buildup of soil pathogens, especially in light of previous research showing that continuous cotton cultivation increases the densities of fungal pathogens in the soil [18].

When interpreting our results, it is important to remain cognizant of the challenges of drawing causal inferences from observational data. The key assumption required to make causal inferences from regression coefficients is that all variables that affect both the treatment assignment (crop rotation, in our analyses) and the response variable (yield and *L. hersperus* density, in our analyses) are included in the model; this ensures that the probability of receiving each treatment becomes, conditional on the predictor variables included in the model, conditionally independent of the response variable [28]. In experimental studies, the treatment assignment is typically controlled by the experimenter, so one can be confident that the only difference between treatment and control groups is in fact the treatment. However, in observational studies, it is impossible to prove definitively that there was no other factor that affected both the treatment assignment and the response variable (thus spuriously suggesting a treatment effect).

As such, we want to be very clear that our hypothesis that the effects of rotation on yield are mediated by effects on *L. hesperus* densities is exactly that - a hypothesis. While our data do support a negative *association* between effects on *L. hesperus* and effects on yield, we cannot prove with observational data that the varying effects on yield are *caused* by the varying effects on *L. hesperus*. This could be a fruitful topic for future experimental work.

Although causality is impossible to prove using observational data, ecoinformatics paves the way for implementing data-driven agricultural strategies and allows us to mine large datasets to explore important questions that are difficult to address experimentally. While by no means a replacement for experimentation, ecoinformatics can be a cost-effective and realistic complementary approach. In particular, our result identifying the effects of crop rotation on *L. hesperus* density would have been extremely difficult to reach experimentally. Since *L. hesperus* readily disperse across spatial scales of more than 1000 meters [37], an experimental study would have required massive plots comparable to the size of commercial fields in order to adequately capture their spatial dynamics.

Our results have numerous practical applications for commercial cotton growers. Growers with knowledge of the crop rotations associated with depressed cotton yield could make more informed decisions, selecting the sequence of crop cultivations that lead to maximized yield. When feasible, cotton plantings could be avoided following crops that decrease cotton yield, and instead limited to fields where crops that increase cotton yield were previously planted. In some cases, market conditions may lead a grower to plant cotton following a yield-depressing crop, even given the knowledge of likely yield loss. In those situations, our results may still be helpful, as an early warning sign of a potential pest problem in a particular field could allow the grower and PCA to focus pest detection efforts on that field and provide time to eliminate the problem before severe yield loss was incurred.

Our results suggest that the yield effects of crop rotational histories in cotton are relatively modest in magnitude: the posterior means for effects of any specific crop were mostly under 15%. However, given the tight profit margins of commercial agriculture, a 15% change in yield could translate into a far greater percentage change in profit, and could therefore be of substantial economic significance to a grower. As we seek to feed a growing worldwide population while doing minimal harm to the environment, crop management practices that increase yield while reducing the need for costly and damaging pesticides and fertilizers are of great value. Crop rotation is one such method, and we are optimistic that ecoinformatics approaches may be helpful in elucidating the details of how to optimally implement crop rotation.
